# Sarcoma undifferentiated (unclassified) fusiform cell type of mesentery: a case report and literature review

**DOI:** 10.11604/pamj.2020.35.58.17749

**Published:** 2020-02-26

**Authors:** Korbi Ibtissem, Korbi Asma, Ennaceur Farouk, Hajji Ahmed, Njima Manel, Boughanmi Faiez, Zouari Khadija, Faleh Raja

**Affiliations:** 1 Department of General and Digestive Surgery, University Hospital of Monastir, Monastir, Tunisia; 2 Department of Gynecology and Obstetrics, University Hospital of Monastir, Monastir, Tunisia; 3 Department of Pathology, University Hospital of Monastir, Monastir, Tunisia

**Keywords:** Case report, sarcoma, undifferentiated (unclassified) fusiform cell type, mesentery

## Abstract

Sarcomas are a heterogeneous group of malignant tumors that come from mesenchymal tissues. Undifferentiated sarcoma represents approximately 20% of soft tissue sarcomas. This entity represents approximately 20% of soft tissue sarcomas. These tumors are subdivided according to their appearance. Morphological in 4 subtypes: pleomorphic cells, fusiform cells, round cells, epithelioids. We report the case of a 72-year-old woman operated for a complicated adnexal tumor, but it turned out that it was sarcoma undifferentiated (unclassified) fusiform cell type of mesentery. It is a rare and a latent tumor. Its diagnosis is histological. Its treatment consists on surgical wide excision if possible. This type of sarcoma has a poor prognosis considering the limited benefits of radio-chemotherapy. Undifferentiated sarcoma type fusiform cells of the mesentery is an exceptional entity. Its diagnosis is difficult. Its treatment is to discuss case by case, surgery is the best option if it is possible. The prognosis is bad. This entity remains to be studied.

## Introduction

Sarcomas are a heterogeneous group of malignant tumors that come from mesenchymal tissues [1]. The term undifferentiated sarcoma is used to refer to malignant mesenchymal tumors that do not meet the criteria for a well-defined histopathological entity [2]. This entity represents approximately 20% of soft tissue sarcomas [1]. These tumors are subdivided according to their appearance. Morphological in 4 subtypes: pleomorphic cells, fusiform cells, round cells, epithelioids [3]. They are considered to be high-grade sarcomas and have a poor prognosis [1]. Sarcomas usually develop at the expense of deep soft tissues of the extremities (50%) and retroperitoneal (15%) and intra-abdominal tissues. Apart from gastrointestinal stromal tumor (GIST), intraabdominal sarcomas represent only (5 to 10%), but mesenteric localization is much rarer [4]. We report the case of an undifferentiated mesenteric sarcoma with fusiform cells.

## Patient and observation

**Figure 1:**
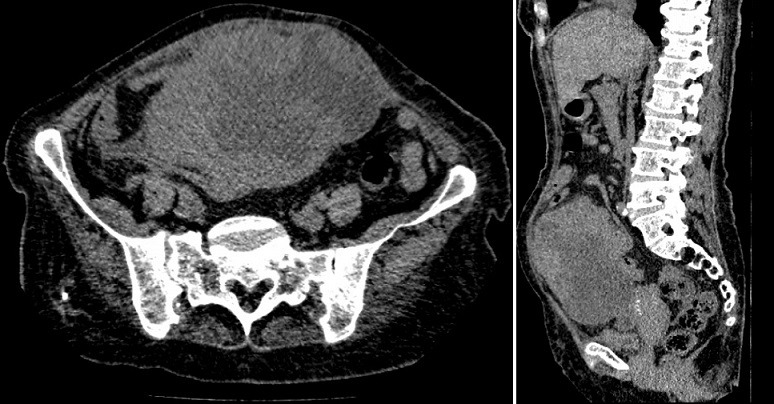
CT aspect of the mass

**Figure 2:**
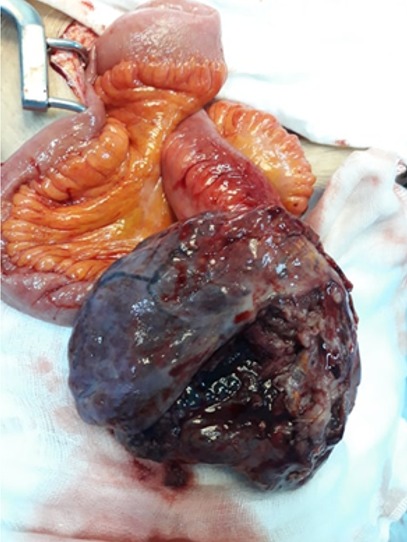
Per operative photo of the mass

**Figure 3:**
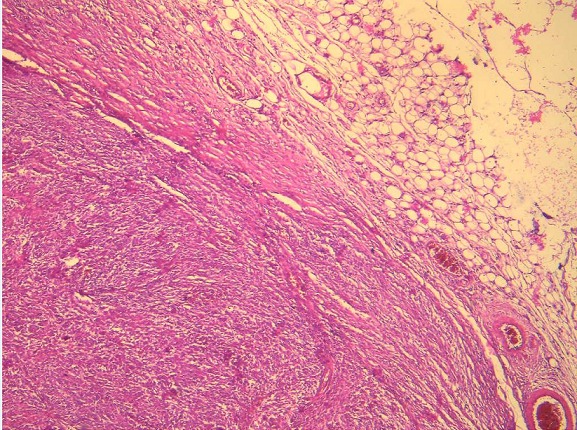
Mesenteric dense cellular proliferation (HEx40)

**Figure 4:**
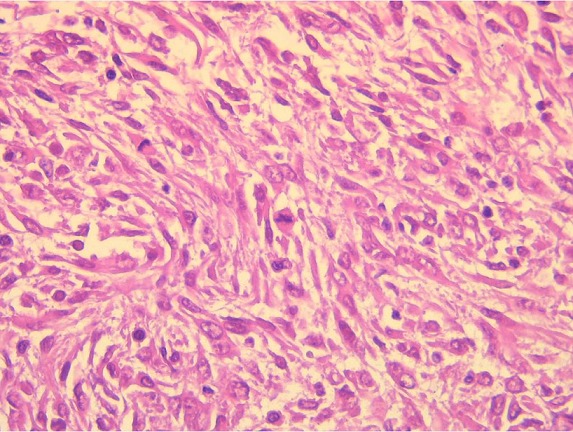
Proliferation formation of spindle cells with moderately nuclear atypia and numerous mitoses (Hex400)

This is a 72-year-old woman with a history of hypothyroidism, hypertension, atrial fibrillation (ACFA), ischemic stroke 3 years ago with left hemiparesis, cavum cancer 1 year ago treated with radiotherapy. She consulted the emergency room for pelvic pain that had been changing for 1 day. On examination we found an afebrile patient, with a PA at 16/08, a pulse at 100 bpm. On abdominal examination we found an abdominopelvic mass of 15*10 cm. A biology Hb = 7.1, GB = 9600, creatinine = 102, urea = 8. Abdominal computed tomography (CT) without contrast injection was reported for renal failure, which showed a pelvic mass of 15*10 cm probably necrotic (Figure 1). The diagnosis of a complicated adnexal tumor was suspected, hence the decision to operate the patient urgently. It was operated medianly. On exploration, we found a mass of 13 cm at the expense of the necrotic small hail adhering to the peritoneum at the level of the pelvis (Figure 2). A 30 cm resection of the small bowel was performed with a small-bowel anastomosis at the mechanical forceps. The patient was transferred to the postoperative unit where she received adequate transfusion and resuscitation. The postoperative course was simple with a dietary recovery at day 5. The patient was discharged 15 days postoperatively. Histopathological examination found spindle-cell type undifferentiated (unclassified) sarcoma with healthy resection margins (Figure 3, Figure 4).

## Discussion

The mesentery is a frequent propagation pathway for malignant tumors through the peritoneal cavity, on the other hand, primary mesenteric tumors are rare and predominantly benign [5]. The most frequent primary mesenteric tumors are desmoid tumors [6]. Other rarer tumors such as sarcomas are also found. Histologically, and according to the 4^th^ edition of the classification of soft tissue and bone tumors of 2013, sarcomas are subdivided into several subtypes according to their differentiation (adipose, fibrohystiocytic, smooth or striated muscle, vascular …) and sarcomas of uncertain differentiation. Recently a new chapter has emerged: that of undifferentiated or unclassified sarcomas [1]. In fact, the diagnosis of undifferentiated sarcoma can only be retained after eliminating all lines of recognizable differentiations [1, 3, 4]. This entity groups together 4 subtypes: cell pleomorphic (formerly called malignant fibrous histiocytoma), round cell, epithelioid, spindle-shaped cells as is the case in our patient [3]. Label a sarcoma as being undifferentiated and specify its type is not so obvious despite the fact that it has an implication in the local treatment of this tumor and an impact on the prognosis [3, 7]. In addition, the interpretation of the pathologist can be significantly influenced by the availability of resources and tools to specify a certain line of differentiation [3, 8] such as context, experience, biopsy size, immunohistochemistry availability, electron microscopy and molecular biology [1, 3, 9]. Patients may present with abdominal pain, an abdomino-pelvic mass of transit disorders, anorexia or weight loss and are rarely complicated by intussusception, perforation or suppuration [10, 11, 12]. Undifferentiated/unclassified sarcomas pleomorphic cells such as fusiform cells affect in particular elderly patients [1, 3]. Round-cell sarcomas are more common in young patients in whom sarcoma associated with translocation must be sought [1, 2, 13]. According to Fletcheret *et al.* [3], the sub classifications of fusiform cell-type sarcomas does not have a great relevance compared to round-cell sarcomas made of their poor prognosis and limited therapeutic options and their low chemo-sensitivity. The only two subtypes of non-fusiform cell-type sarcomas for which there may be significant therapeutic involvement are: monophasic synovial sarcoma, sensitive to chemotherapy (ifosfamide), and the Gleevec-sensitive fibrosarcomatous variant (in the context of local disease) or metastatic) [3]. Concerning the diagnosis, the CT scan can be useful in providing the tissue characteristics of tumors and its reports of a mesenteric tumor [6]. According to Faizi *et al*. magnetic resonance imaging (MRI) is the most performing in the diagnosis of mesenteric sarcomas with the possibility of predicting the benign or malignant nature of tumors and even the histological type and positron-emission tomography (PET) scan finds a place in the context of sarcomas with ganglionic extension on MRI [14]. As any intra-abdominal sarcoma, the diagnosis is histological. In cases where sarcoma is locally limited, non-metastatic or with resectable liver metastases, treatment is surgical wide excision with healthy margins is the rule unlike dementia or with unresectable metastases or curative surgery is no longer an option with an unfortunate prognosis considering the limited benefits of radio-chemotherapy [4, 15].

## Conclusion

Undifferentiated or unclassified sarcoma is a new entity that accounts for 20% of soft tissue sarcomas and 25% of irradiated tissue sarcomas. Its mesenteric localization is rare. In this context the undifferentiated sarcoma type fusiform cells of the mesentery is an exceptional entity. Its diagnosis is difficult. Its treatment is to discuss case by case, surgery is the best option if it is possible. The prognosis is bad. This entity remains to be studied.

## Competing interests

The authors declare no competing interests.
